# An International Society and Journal for Equity in Health: 10 years on

**DOI:** 10.1186/1475-9276-10-11

**Published:** 2011-02-27

**Authors:** John Furler, Elizabeth Harris, Fran Baum, Jane Dixon, Angela Lawless, Daniel Maceira, Lexi B Nolen, Barbara Starfield

**Affiliations:** 1 Department of General Practice, University of Melbourne, Melbourne, Australia; 2 Centre for Primary Health Care and Equity, University of New South Wales, Sydney, NSW, Australia; 3 Southgate Institute of Health, Society and Equity, Faculty of Health Sciences, Flinders University, Adelaide, Australia; 4 National Centre for Epidemiology and Population Health, Australian National University, Canberra, Australia; 5 South Australian Community Health Research Unit, Flinders University, Adelaide, Australia; 6 Center for the Study of the State and Society (CEDES), Buenos Aires, Argentina; 7 Center to Eliminate Health Disparities, Global Health Program, University of Texas Medical Branch, Galveston, TX, USA; 8 Johns Hopkins Bloomberg School of Pubic Health, Baltimore, MD, USA

## 

June 2010 marked the 10th Anniversary of the foundation meeting of the International Society for Equity in Health (ISEqH). The formation of the Society was a bold statement, with ambitions to be a global body "to promote equity in health and health services internationally through education, research, publication, communication and charitable support"[1]. The Society particularly aimed to be an organisation that facilitated research on how better to understand and address inequities in health. The main activities of the Society have been a series of biannual conferences as well as the establishment of the International Journal for Equity in Health, the official (but independent) publication of the Society. This paper sets out to record some of the milestones of the Society drawing on the reflections of key researchers who attended the conferences as well as others. The history of the Society will help shape its future and how it responds to important issues facing all interested in global efforts to address continuing and unacceptable inequities in health.

## Establishing ISEQH and THE 1^ST ^MEETING IN CUBA 2000

The first meeting was important in setting the tone of the Society. The late 1990s saw a world-wide renaissance in attention to the socio-economic determinants of health and the sources of unjust differences in health outcomes. It was in the context of this growing evidence that Barbara Starfield approached colleagues in 1999 seeking help in establishing a global organisation devoted to the science of health equity, reasoning that international societies existed for health economics, medical services and public health, but not equity.

Together with Jose Maria Paganini from Argentina, Barbara had been working on the formation of the society for 3 years, approaching others with an aim of using science to address inequitable health status. Jane Dixon (co-ordinator of the Australian Health Inequalities Research Collaboration) provided early assistance in crafting an interim constitution for the Society appealing to an international membership and potential donors. These early efforts were successful in attracting grants to support the first conference, especially the participation of developing world delegates.

In light of a US trade embargo and its international endorsement, the decision to hold the first conference in Cuba was both bold and controversial, but it made enormous sense given the country's reputation for running a First World health and education system, and with health outcomes and health differences the envy of many OECD countries. Cuba exemplified the evidence of the time that per capita income of US$2500 was sufficient to provide good health status and that anything more than this provided only marginal improvements.

The inaugural conference was held in Havana in June, 2000. 210 people attended, coming from 32 countries. One third of delegates came from the US, while Argentina and Australia had 26 and 20 delegates respectively. Sizable contingents came from Sweden and the United Kingdom.

For most participants, the prospect of the meeting was enormously exciting: the majority had not been to Cuba before and mystery surrounded its somewhat demonic reputation (particularly in the US) on the one hand and its excellent health system on the other;. The pre-conference field trips to primary health care and clinical settings provided vivid illustrations of a sophisticated health strategy; walking through the old city full of derelict housing were good reminders of the effect of the US embargo.

The quality of conference printed materials and the support from the Executive Committee made this an inclusive scientific society. The choice of welfare economist, Sudhir Anand as keynote speaker was very well received. Intellectual and ideological arguments prevailed in some sessions, particularly over the relationship of equity to observed social gradients in health status, and the extent to which the Society should embrace political advocacy (which was in fact precluded by the legal requirements of non-profit status which in turn was required to obtain foundation support). The interim executive maintained a unified position that this was a scientific body. The definition of inequity that was adopted was landmark, because it was the first to lend itself to measurement: 'the SYSTEMATIC and potentially remediable differences in one or more aspects of health across populations or population groups defined demographically, socially, or geographically.'

A Scientific Committee of 20 researchers was appointed spanning Taiwan, Trinidad, India, Mexico, South Africa, the Netherlands and others, The first conference was a success: membership doubled in 18 months from an initial 125 people in 2000. That meant membership fees could augment the halving of Foundation grants (the first Budget tabled in Cuba showed these amounted to $110,000). The Argentinean Society of Physicians offered to continue to host the secretariat and three newsletters were issued in and around the first conference. To ensure good communications in the lead up to the next conference planned for Toronto, the location of a small part-time secretariat to assist the committee shifted in 2001 to that city and continued to work to support the organisation over the past decade.

## Subsequent conference

Subsequent conferences were held in Toronto (2002), Durban (2004), Adelaide (2006) and Crete (2009). The Toronto Conference had the theme "Research in the Service of Policy and Advocacy for Health and Health Services". Key international health equity researchers attended form developed and developing economies. The Conference also aimed to facilitate the development of a Canadian Health Equity Network. In Durban the conference had a theme of *Pathways to Equity in Health: Using Research for Policy and Advocacy*. The conference brought together participants from more than 50 countries from six continents. Importantly, the conference was book-ended by regional meetings of the convening organisations, GEGA (the Global Equity Gauge Alliance) and EQUINET (the Regional Network for Equity in Health in Southern Africa), allowing participants to attend all three conferences and a variety of workshops over a 9 day period. This highly effective collaboration strengthened synergy and cross-cultural understanding of challenges and strategies among researchers, policy makers and managers. The high level of participation by African researchers and students was not only a result of the location of the conference but also of strong cooperation between the hosting organizations to maximize the equitable distribution of funds to participants. Over 40 low- and middle-income countries were represented among participants, including over a dozen African countries.

The 2006 Conference in Adelaide had the theme of "Creating Healthy Societies through Inclusion and Equity". It attracted over 230 participants from more than 35 countries. Indigenous health was a major focus of the conference and a second complementary theme examined the use of arts and community cultural development in promoting health and equitable outcomes. The Scientific Committee included strong representation from Indigenous, Maori and Pacific health researchers. Again low to middle income countries were well represented with 37 countries represented and approximately 40% of the presenters coming from low-to-middle income countries. There were over 25 presentations on Aboriginal health as well as a full-day workshop on Indigenous Health. The conference brought together a diverse set of researchers, both Indigenous and non-Indigenous, community health practitioners, including traditional medicine practitioners, community leaders, artists, and policymakers.

In 2009 a conference was held in Crete. Sponsored by the Greek School of Public Health in partnership with the Canadian Society for International Health, the organisers had initially found it difficult to attract funding and this significantly reduced the number of participants from low and middle income countries. This experience highlighted the importance of a strong organising committee with local knowledge and the importance of providing basic support for meals and travel if a broad base of researchers is to be attracted.

Challenges for a small society organising global meetings have been in maintaining a high level of academic scholarship, but also in moving from descriptive research to more normative research around policy analysis, advocacy needs and strategies cooperative planning and intervention research. Despite these challenges the meetings have been important networking opportunities for furthering research in the area of equity in health. The development and launching of the World Health Organisation (WHO) Commission on Social Determinants of Health (CSDH) and the 2008 release of the WHO report on Primary Health Care provided an exciting context for the latest meeting, raising hopes of action on health equity in a practical sense through governmental and non-governmental policy and action. WHO used the conferences as one means to publicise the planning and then work of the CSDH.

## Establishing the Journal

Formation of the International Journal for Equity in Health has been a critical linked activity, keeping alive the aims of the society. Nevertheless this has not been without challenges. Shortly after the formation of the International Society for Equity in Health, Fiona Godlee (who had been with the British Medical Journal and who had recently participated in the development of the open-access Biomedical Central (BMC) journals) approached Barbara Starfield with the idea to develop a journal devoted to equity in health. At the initiation of BMC, access to articles would be free on the web and without any charge to authors. The invitation was irresistible because it was consistent with the mission of the society, which was primarily to encourage research in equity in health among individuals without previous experience in this area, and from researchers and countries underrepresented in other journals. Free access to publication by new researchers as well as free access to readers (to foster the spread of knowledge about equity in health) seemed to be just what the Society needed. Unfortunately, shortly after the formation of the journal, the for-profit publisher instituted author charges, thus undermining the goal of increasing access to publication by new researchers who had less ability than grant-supported researchers to pay for their publications. It also compromised the journal's ability to attract publications from more seasoned and well-known researchers who are able to publish their work in more prestigious journals at no cost. The editors were able to negotiate for a small proportion of accepted papers to be exempt from author charges because the authors were from low income countries, but the policy of charging for publication acted as a barrier.

Despite limitations (limited editorial staff and heavy workload recruiting reviewers which precluded more proactive development), the journal has continued to grow and receive attention in the field of equity in health. The review process is rigorous, involving at least two peer reviewers as well as editorial input into decisions and revision and acceptance of papers. In the approximately ten years of its existence, the number of submissions and acceptances has grown steadily. At the present time, about 57% of submitted articles are accepted; this relatively high rate reflects a very active peer reviewer/author interaction as the goal is to work with authors to revise papers until they are acceptable for publication. Of the ten most cited papers during April 2010 (the latest data available), 6 were from developing countries. The high salience of the work is evident from the number of publications that continue to be cited even years after their publication; in April 2010, two of the ten most cited papers were published before 2007. As of 2011, the journal will be fully included in all impact rating systems, which only occurs when a journal has sufficient peer-reviewed publications to merit such ratings. Preliminary impact ratings for the journal are approximately the same as those for similar (health services research) BMC journals. This inclusion in the impact rating system has occurred while the journal has continued to maintain wide representation from non-Western countries.

The focus for the journal will continue to be in encouraging the publication of health equity research, particularly with authors from countries under-represented in equity research publications. The editorial group hopes to find a way carry out even more innovative activities than has been possible under very constrained conditions.

## Where to from here?

The energy of the early conferences and the achievements of the journal in its short life are to be celebrated and nurtured. The conferences have provided a meeting point where most of the key researchers in health equity from across the world have attended at some time. In particular the conferences and the journal have both provided a voice to researchers from low and middle income countries, giving life and embodiment to the values of inclusion, action-based research, research-to-policy processes, and the vital role of civil society in strengthening action for health equity.

The importance of achieving health equity has been placed firmly on international agenda by the publication in 2008 of the report of the Commission on the Social Determinants of Health. The Commission was able to draw on the expertise of members of ISEqH through the work of its nine knowledge networks. Implementation of the CSDH's findings will continue to need the support of societies such as ISEqH.

Despite this international context the society and journal both face challenges, not least the constant need to raise funds in a tightening philanthropic and Government climate. Without funds the society and journal continue to rely on the freely given time of a small number of individuals. Marrying diverse perspectives and articulating a common purpose and vision is a challenge for any group with a global base and mission. This is particularly so for a member based organisation in an era of many public health societies, all of which make demands of time and money on members. Nevertheless the global perspective of the Society remains a core value.

Ten years on, much has been achieved. The Society has played a role in supporting the wider group of researchers, policymakers and practitioners with a commitment to social justice and equity. There is still more to do. The current Board has expressed interest in focusing on building research capacity in low and middle income countries in the next two years. The Society is particularly keen on ensuring that health reform is closely linked to a focus on equity. The sixth International Conference of the Society is planned for Sept 26-28 in Cartagena Colombia and will have as a theme "Making policy a health equity building process".

## Competing interests

DM is President of the ISEqH and JF, EH and LN are members of the Executive of the ISEqH (all positions are unpaid). BS is Editor-in-Chief and JF is an Associate Editor of the IJEqH (unpaid)

## Authors' contributions

EH conceived of the paper. All authors contributed sections to the paper. All authors read and approved the final version.
